# *Angiostrongylus cantonensis* Infection in Brown Rats (*Rattus norvegicus*), Atlanta, Georgia, USA, 2019–2022

**DOI:** 10.3201/eid2910.230706

**Published:** 2023-10

**Authors:** Nicole L. Gottdenker, Rafael Antonio Nascimento Ramos, Hassan Hakimi, Brittany McHale, Sam Rivera, Bryce M. Miller, Elizabeth W. Howerth, Caitlin E. Burrell, Justin M. Stilwell, Rita McManamon, Guilherme G. Verocai

**Affiliations:** University of Georgia College of Veterinary Medicine, Athens, Georgia, USA (N.L. Gottdenker, B. McHale, B.M. Miller, E.W. Howerth, C.E. Burrell, R. McManamon);; Texas A&M University School of Veterinary Medicine and Biomedical Sciences, College Station, Texas, USA (R.A.N. Ramos, H. Hakimi, G.G. Verocai);; Zoo Atlanta, Atlanta, Georgia, USA (S. Rivera);; Mississippi State University College of Veterinary Medicine, Starkville, Mississippi, USA (J.M. Stilwell)

**Keywords:** Angiostrongylus cantonensis, parasites, nematodes, zoonoses, eosinophilic meningoencephalitis, vector-borne infections, rodents, brown rats, Rattus norvegicus, United States

## Abstract

Rat lungworm (*Angiostrongylus cantonensis*)*,* a zoonotic parasite invasive to the United States, causes eosinophilic meningoencephalitis. *A. cantonensis* harbors in rat reservoir hosts and is transmitted through gastropods and other paratenic hosts. We discuss the public health relevance of autochthonous *A. cantonensis* cases in brown rats (*Rattus norvegicus*) in Atlanta, Georgia, USA.

Rat lungworm, *Angiostrongylus cantonensis* (Strongylida: Metastrongyloidea), causes eosinophilic meningoencephalitis (neural angiostrongyliasis) in humans and other accidental mammal hosts. This vectorborne nematode has an indirect life cycle in which several rodent species, including *Rattus* spp., serve as definitive hosts (*1*). Rodents become infected by ingesting terrestrial gastropods acting as intermediate hosts infected with third-stage larvae (L3). In the rodent host, L3 migrate through vasculature to the central nervous system and after 2 molts become adult nematodes that migrate to the pulmonary artery (*1*). After mating, females lay eggs that hatch first-stage larvae (L1) in lung airspaces. L1 ascend the trachea, pass into the digestive system after being swallowed by the host rat, and exit the body through feces (*1*). Subsequently, gastropods ingest nematode L1 after which the larvae develop to the infective L3 stage. Paratenic hosts, such as fish, frogs, and crustaceans, can also harbor *A. cantonensis* L3, which can be transferred to rodents and accidental hosts (*2*). 

*A. cantonensis*, originally described in Asia, where most human infections are reported, is now endemic in different regions of the world (*2*). In the United States, *A. cantonensis* was initially reported in Hawaii (*3*), and later in Texas, Louisiana, Alabama, and Florida, likely introduced by infected rats and gastropods through trade routes, such as on merchant ships (*3*–*7*). We confirm autochthonous *A. cantonensis* infection in brown rats (*Rattus norvegicus*) in Atlanta, Georgia, USA, and briefly discuss the relevance of these findings to human and animal health. 

## The Study 

We collected tissue samples (brain, heart, liver, kidney, lung, spleen, skeletal muscle, skin, gastrointestinal tract, adrenal gland, and gonads) from 33 wild brown rats found dead during 2019–2022 on the grounds of a zoological facility located in Atlanta, Fulton County, Georgia (33°44′1.536″N; 84°22′19.416″W). We stored samples in 10% neutral buffered formalin and processed them for routine histopathologic evaluation as part of opportunistic monitoring of wildlife found dead on zoo grounds. Of the rats we histologically evaluated, 7/33 (21.2%) had nematodes in heart, pulmonary artery, and brain tissues (Table; Figure). 

**Table:** Histopathologic findings of *Angiostrongylus cantonensis* nematode infection and molecular confirmation in brown rats (*Rattus norvegicus*), Atlanta, Georgia, USA, 2019–2022*

ID, age class/sex	Case submission date	Histopathologic findings	GenBank accession no.
Case 1, adult/M	2019 Feb 19	Brain: hemorrhagic and lymphohistiocytic meningoencephalitis with intralesional nematodiasis (adult nematodes, presumptive *Angiostrongylus* spp.)	OQ793715
Case 2, adult/F	2021 Feb 12	Lung: severe multifocal chronic nodular nematodiasis (adult nematodes and larvated ova, presumptive *Angiostrongylus* spp.)	OQ793716
Case 3, juvenile/ unknown	2021 Jan 20	Heart: intravascular (i.e., cardiac chamber, pulmonary artery) nematodiasis	NA
Case 4, Age unknown/unknown	2021 Aug 24	Lung: pulmonary arterial nematodiasis; pulmonary hemorrhage and edema	NA
Case 5, adult/unknown	2022 Apr 25	Heart and pulmonary artery: cardiac nematodiasis with endothelial pulmonary aortic subendothelial myxomatous change Lung: nematode cross section with similar characteristics to heart nematodes	NA
Case 6, adult/unknown	2022 Aug 09	Heart and pulmonary artery: intraventricular and intra-arterial nematodiasis Lung: moderate intraluminal, peritracheal, and pulmonary hemorrhage	OQ793717
Case 7, adult/M	2022 Oct 18	Lung: eosinophilic pulmonary arteritis with degenerate intraluminal nematodes	OQ793718

*Pre- or postmortem predation of some rats might have occurred before rats were found. Thus, representative samples of all organs might not have been available for evaluation and in some cases, sex or age class could not be determined. ID, identification; NA, not available.

**Figure:**
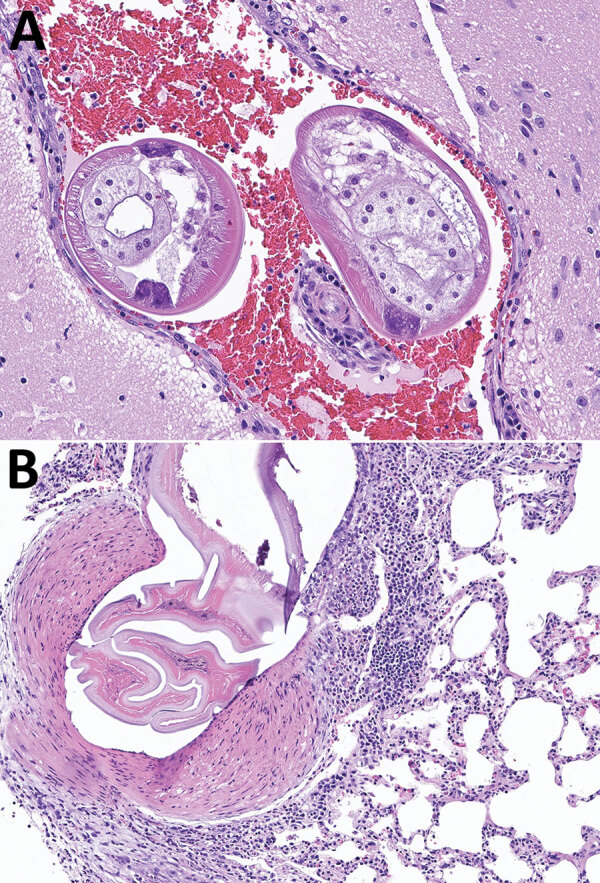
Brain and lung tissue samples showing *Angiostrongylus cantonensis* infection in brown rats (*Rattus norvegicus*), Atlanta, Georgia, USA, 2019–2022. A) Representative tissue section from the brain, stained with hematoxylin and eosin. The meninges and ventricles are multifocally and moderately expanded by abundant hemorrhage. Within the meninges and ventricles, occasional cross sections through nematodes can be seen. Nematodes were ≈250–300 μm in diameter with a thin eosinophilic cuticle, pseudocoelom, polymyarian coelomyarian musculature, lateral chords, and multinucleated intestine. Original magnification ×200 μm. B) Representative tissue section from the lung stained with hematoxylin and eosin. A large pulmonary artery contains fragments of a degenerative nematode characterized by a thin eosinophilic cuticle, pseudocoelomic space, and polymyarian coelomyarian musculature. The subtending arterial wall was sometimes necrotic and variably infiltrated by eosinophils, lymphocytes, and macrophages. The vessel also displays hypertrophy of the tunica media and occasional hypertrophy of the endothelial cells. Original magnification ×100 μm.

Where intravascular nematodes were observed, we extracted genomic DNA from paraffin-embedded tissue sections using QIAamp DNA FFPE Tissue Kit (QIAGEN, https://www.qiagen.com) according to manufacturer recommendations. PCR reactions targeted a 200-bp segment of the mitochondrial cytochrome oxidase subunit 1 gene (*cox1*). We performed a 25 μL reaction containing 0.25 μmol each of primers CO1ACF7 (5′-TGCCTGCTTTTGGGATTGTTAGAC-3′) and CO1ACR7 (5′-TCACTCCCGTAGGAACCGCA-3′), 1× GoTaq Green Master Mix (Promega Corporation, https://www.promega.com), and 2.5 μL of DNA template (*8*). Cycling procedure involved initial denaturation at 95°C for 2 min, then 40 cycles at 95°C for 30 s, 50°C for 30 s, 72°C for 90 s, and a final extension at 72°C for 5 min. We used nuclease-free water as a negative control and DNA of *Dirofilaria immitis* as a positive control. We purified PCR products using the EZNA Cycle Pure Kit (OMEGA Bio-Tek, https://www.omegabiotek.com) according to manufacturer instructions. We aligned and compared generated sequences with homologous *A. cantonensis* sequences available in GenBank (https://www.ncbi.nlm.nih.gov/genbank). We determined genetic distances and performed phylogenetic analysis using MEGA X 10.1 (*9*) (Table). 

Our molecular analysis confirmed the identity of *A. cantonensis* in 4/7 samples that had nematodes visible on histologic examination of heart, pulmonary artery, and brain tissues (Table). All 4 sequences were 100% identical to each other and to *A. cantonensis* sequences belonging to haplotype 17a, previously reported from Louisiana, USA. Among homologous sequences available in GenBank from *A. cantonensis* isolates from the United States, those belonging to haplotype 17b (Louisiana and California) were 99.5% similar, haplotype 8b (Louisiana) 98.9% similar, and haplotype 5a (Hawaii) 98.9% similar to those in haplotype 17a. Overall, compared with other *A. cantonensis* haplotypes included in the phylogenetic analysis, similarity of sequences ranged from 93.1%–99.5%, clustering in a clade with 86% bootstrap support (Appendix).

## Conclusions

Discovery of autochthonous cases of *A. cantonensis* infection in definitive host rodents collected during 2019–2022 in the state of Georgia, suggests that this zoonotic parasite was introduced to and has become established in a new area of the southeastern United States. Although we molecularly confirmed diagnosis in only 4/7 cases, the remaining rats had intravascular nematodes morphologically consistent with *A. cantonensis* and typical associated lesions*.* We could not molecularly confirm the remaining 3 cases because of insufficient sample quality and DNA degradation; thus, we could not rule out the presence of other nematode species. 

Because *A. cantonensis* lungworm previously was identified in rats in neighboring states Florida and Alabama, *A. cantonensis* populations likely were in Georgia much earlier than 2019, when the first positive rat was identified in Atlanta. Furthermore, 6 suspected autochthonous human angiostrongyliasis cases were detected during 2011–2017 in Texas, Tennessee, and Alabama (*10*). Among captive wildlife, *A. cantonensis* lungworm has been reported in nonhuman primates in Florida (*11*), Louisiana (*7*,*12*), Texas (*4*), and Alabama (*13*), and a red kangaroo in Mississippi (*14*). Among free-ranging wildlife native to the southeastern United States, *A. cantonensis* infections have been identified in armadillos and an opossum (*15*). 

Various native and exotic gastropod species have been shown, both naturally and experimentally, to be susceptible intermediate hosts (*3*,*5*,*11*). Although details of *A. cantonensis* invasion and spread are not fully known, identification of introduced gastropods as intermediate hosts (*11*) and Cuban tree frogs as paratenic hosts (*6*) in the southern United States suggest anthropogenic disturbance and climate-induced change in local food webs might be amplifying *A. cantonensis* transmission. Clearly, *A. cantonensis* lungworm in urban rat populations, gastropod intermediate hosts, and other paratenic hosts in the populous greater Atlanta area pose a possible threat to the health of humans and domestic, free-ranging, and captive animals. 

Understanding patterns of historic, contemporary, and future expansion of the range of *A. cantonensis* lungworm in North America through surveillance, genetic analysis, and modeling is critical to mitigating risk to humans and other animals for infection by this parasitic nematode, which harbors in synanthropic wild rodent and intermediate host populations. Medical and veterinary professionals throughout the southern United States should consider *A. cantonensis* infection in differential diagnoses of aberrant central nervous system larva migrans, eosinophilic meningitis, and meningoencephalitis. 

Appendix Additional information on study of *Angiostrongylus cantonensis* infection in brown rats, Atlanta, Georgia, USA.
