# Supplementary material for: Angiostrongylus cantonensis Infection in Brown Rats (Rattus norvegicus), Atlanta, Georgia, USA, 2019–2022
Source: Emerg Infect Dis. 2023 Oct;29(10):2167–70. doi: 10.3201/eid2910.230706 (PMC10521602; doi:10.3201/eid2910.230706)
Supplement: Appendix — Additional information on study of Angiostrongylus cantonensis infection in brown rats, Atlanta, Georgia, USA. [file 23-0706-Techapp-s1.pdf]

Article DOI: https://doi.org/10.3201/eid2910.230706

EID cannot ensure accessibility for supplementary materials supplied by authors. Readers who have difficulty accessing supplementary content should contact the authors for assistance.

Angiostrongylus cantonensis Infection in Brown Rats (*Rattus norvegicus*), Atlanta, Georgia, USA, 2019–2022

Appendix

Evolutionary Analysis by Maximum-Likelihood Method

The evolutionary history was inferred by using the maximum-likelihood method and Hasegawa- Kishino-Yano model (*1*). The bootstrap consensus tree inferred from 1,000 replicates (*2*) is taken to represent the evolutionary history of the taxa analyzed (*2*). Branches corresponding to partitions reproduced in less than 50% bootstrap replicates are collapsed. The percentage of replicate trees in which the associated taxa clustered together in the bootstrap test (1,000 replicates) are shown next to the branches (*2*). Initial trees for the heuristic search were obtained automatically by applying Neighbor-Join and BioNJ algorithms to a matrix of pairwise distances estimated using the Maximum Composite Likelihood (MCL) approach, and then selecting the topology with superior log likelihood value. The rate variation model allowed for some sites to be evolutionarily invariable ([+I], 68.94% sites). This analysis involved 43 nucleotide sequences. Codon positions included were 1st+2nd+3rd+Noncoding. There was a total of 190 positions in the final dataset. Evolutionary analyses were conducted in MEGA X (*3*).

References

1. Hasegawa M, Kishino H, Yano T. Dating of the human-ape splitting by a molecular clock of mitochondrial DNA. J Mol Evol. 1985;22:160–74. PubMed https://doi.org/10.1007/BF02101694

2. Felsenstein J. Confidence limits on phylogenies: an approach using the bootstrap. Evolution. 1985;39:783–91. PubMed https://doi.org/10.2307/2408678

3. Tamura K. MEGA X: molecular evolutionary genetics analysis across computing platforms. Mol Biol Evol. 2018;35:1547–9. PubMed https://doi.org/10.1093/molbev/msy096

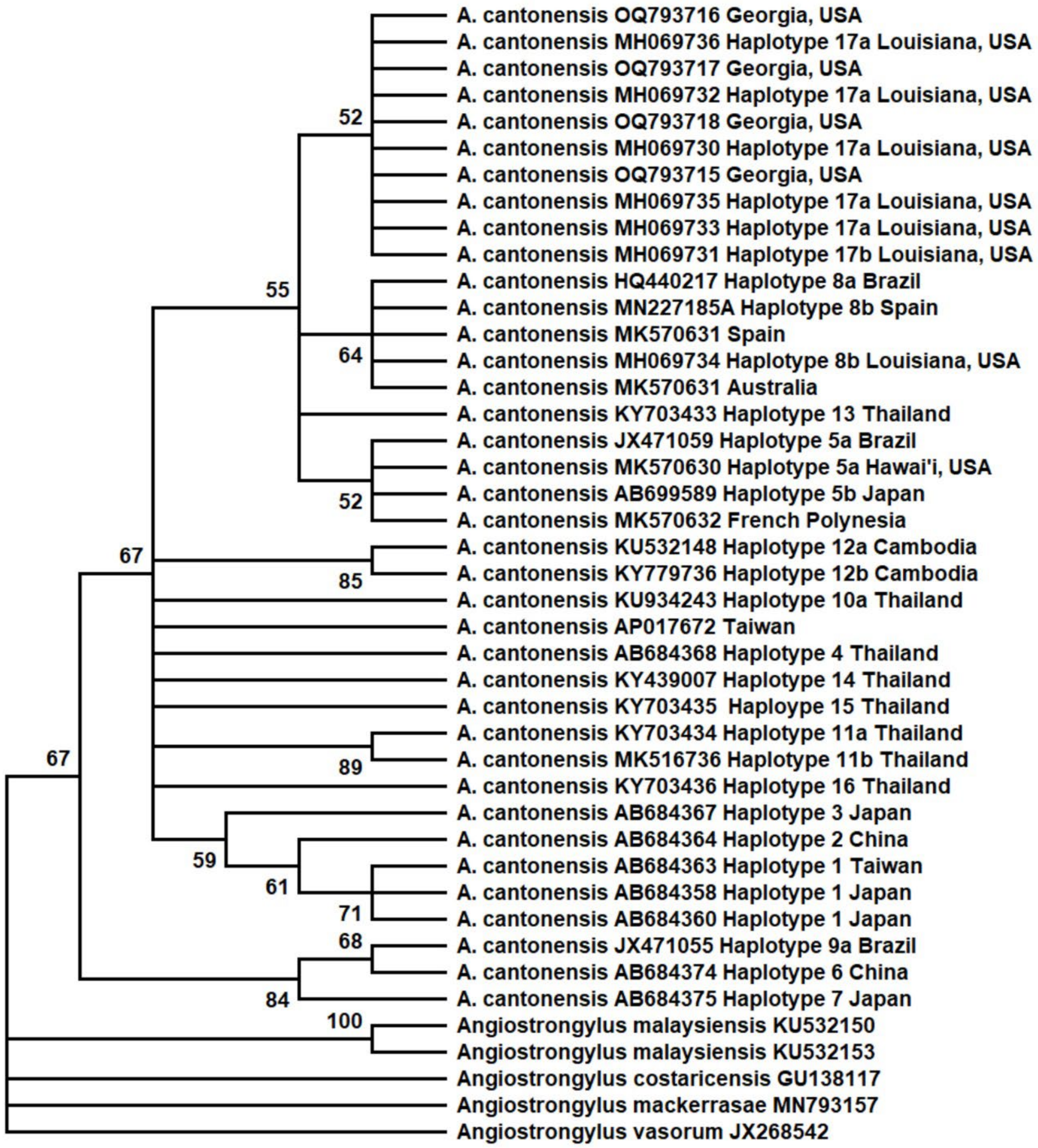

Appendix Figure. Maximum-likelihood phylogenetic tree (1,000 bootstrap replicates) depicting the phylogenetic relationships of *Angiostrongylus cantonensis* sequences generated from rat samples from Atlanta, Georgia, USA, and other representative sequences of various *A. cantonensis* haplotypes from various parts of the world.
